# Simplifying Genotyping of Mutants from Genome Editing with a Parallel qPCR-Based iGenotype Index

**DOI:** 10.3390/cells13030247

**Published:** 2024-01-29

**Authors:** Liezhen Fu, Shouhong Wang, Lusha Liu, Yuki Shibata, Morihiro Okada, Nga Luu, Yun-Bo Shi

**Affiliations:** 1 Section on Molecular Morphogenesis, National Institute of Child Health and Human Development, National Institutes of Health, Bethesda, MD 20892, USA; ful@mail.nih.gov (L.F.); wangsh@cib.ac.cn (S.W.); lsliu01@163.com (L.L.); yuki-shibata@nms.ac.jp (Y.S.); morihiro.okada@riken.jp (M.O.); nga.luu@nih.gov (N.L.); 2 Chengdu Institute of Biology, Chinese Academy of Sciences, Chengdu 610041, China; 3 College of Fisheries, Huazhong Agricultural University, Wuhan 430070, China; 4 Department of Biology, Nippon Medical School, Tokyo 180-0023, Japan

**Keywords:** genome editing, genotyping, parallel qPCR, iGenotype index, *Xenopus tropicalis*

## Abstract

Targeted genome editing is a powerful tool in reverse genetic studies of gene function in many aspects of biological and pathological processes. The CRISPR/Cas system or engineered endonucleases such as ZFNs and TALENs are the most widely used genome editing tools that are introduced into cells or fertilized eggs to generate double-strand DNA breaks within the targeted region, triggering cellular DNA repair through either homologous recombination or non-homologous end joining (NHEJ). DNA repair through the NHEJ mechanism is usually error-prone, leading to point mutations or indels (insertions and deletions) within the targeted region. Some of the mutations in embryos are germline transmissible, thus providing an effective way to generate model organisms with targeted gene mutations. However, point mutations and short indels are difficult to be effectively genotyped, often requiring time-consuming and costly DNA sequencing to obtain reliable results. Here, we developed a parallel qPCR assay in combination with an iGenotype index to allow simple and reliable genotyping. The genotype-associated iGenotype indexes converged to three simple genotype-specific constant values (1, 0, −1) regardless of allele-specific primers used in the parallel qPCR assays or gene mutations at wide ranges of PCR template concentrations, thus resulting in clear genotype-specific cutoffs, established through statistical analysis, for genotype identification. While we established such a genotyping assay in the *Xenopus tropicalis* model, the approach should be applicable to genotyping of any organism or cells and can be potentially used for large-scale, automated genotyping.

## 1. Introduction

Reverse genetic manipulation is an important approach, complementary to transgenic studies, for studying gene function and the underlying molecular mechanisms in many biological and pathological processes. Targeted genome editing is a powerful tool in reverse genetic studies. Over the past few decades, multiple strategies have been established to achieve targeted genome editing. Among them include CRISPR/Cas and engineered endonucleases, such as Zinc Finger Nuclease (ZFN) [1] and Transcription Activator-Like Effector Nucleases (TALENs) [2,3,4,5,6,7,8,9,10,11,12,13,14,15]. Genome editing has been successfully applied to diverse organisms including animal models such as *Xenopus tropicalis* [16,17,18] and zebrafish [3,4,5,6,7,8,19,20]. The molecular basis of these genome-editing systems is to guide a nuclease to a target region, either through engineered DNA-binding domains in the cases of engineered endonucleases or designed guide RNAs in the case for the CRISPR/Cas system [3,13], to cause double-strand DNA breaks within the targeted region. The double-strand DNA breaks would trigger the cellular repair system to repair DNA damages either through homologous recombination, which requires homologous DNA templates in the proximity of the target region, or through non-homologous end joining (NHEJ), which often introduces point mutations or nucleotide insertions or deletions (indels) to the targeted region due to its error-prone repair mechanism. When the NHEJ occurs, the newly introduced out-of-frame mutations would disrupt endogenous protein expression through scrapping protein sequences via out-of-frame translations, thus effectively disrupting gene function.

Targeted genome editing often generates various mutations with different efficiencies in the F0 generation (obtained immediately after genome editing) [21,22]. To determine the mutation spectrum and efficiency, various strategies have been developed to genotype the resulting organisms [23], such as the Surveyor assay [24], a two-color fusion protein assay [21], indel detection by amplicon analysis (IDAA) through automated capillary electrophoresis [25], high-resolution melt analysis (HRMA) [26], next-generation sequencing (NGS) [9], and DNA cloning and sequencing analysis. For establishing mutant animal models, the resulting F0 genome-edited organisms with high mutation spectrum and rates would be selected to produce germline mutant animals with a single type of mutation, which is eventually determined by DNA sequencing [27]. For functional studies using such germline animals, effective genotyping is critical but proved challenging, especially in large scale and for organisms with point mutations or short indels. While the screening methods for F0 generation organisms can also be used for genotyping individual germline organisms with point mutations or short indels, they are typically time-consuming and/or inaccurate. Allele-specific PCR detection could be promising approaches to genotype those germline animals, the major challenge was to optimize the specificity of the allele-specific primers with limited selection of primers within the targeting regions of small differences in nucleotide sequences between the wild-type and mutant alleles [28]. We previously established a competitive PCR with dual fluorescent primers to enhance the specificity and reproducibility of genotyping assay [28]. To expand the application of PCR-based genotyping, particularly toward large-scale genotyping, we have now developed a novel approach by using a parallel qPCR analysis-based iGenotype index to genotype organisms with point mutations or short indel mutations. By using the anuran *Xenopus tropicalis* as an example, we show that this simple and easy-to-use method accurately and efficiently genotypes animals with short indels.

## 2. Materials and Methods

### 2.1. Experimental Animals

Wild-type adult *X. tropicalis* frogs were purchased from NASCO. Embryos and tadpoles were generated as described [17]. All animal care and treatments were performed as approved by the Animal Use and Care Committee of the *Eunice Kennedy Shriver* National Institute of Child Health and Human Development of the National Institutes of Health.

### 2.2. Gene Editing and Germline Breeding

Short-guide RNA (sgRNA) for guiding CRISPR/Cas9 to genome targets was designed as previously described [17] to target the exon 1 of *X. tropicalis MBD3* [28] and the exon 2 of *X. tropicalis* thyroid hormone receptor beta gene (*TRβ*) [27], respectively. For *MBD3* knockout, wild-type fertilized eggs were injected with CRISPR/Cas9 mRNA and sgRNA mix at the one-cell stage, screened for the tadpoles with high rates of out-of-frame mutations as described [21], and reared to adulthood (F0 generation frogs). Sexually mature F0 frogs were used one at a time to mate with a wild-type frog to produce F1 generation tadpoles to obtain heterozygous mutant tadpoles containing out-of-frame mutations within the targeted exon [21]. The mutant animals with an 8-nucleotide deletion (MBD3-KO(∆8)) were further confirmed by PCR-cloning the target region and sequencing and reared to adulthood (F1 heterozygous frogs) [28]. A pair of F1 MBD3-KO(∆8) heterozygous frogs were mated to produce F2 generation tadpoles. Animals with a heterozygous *TRβ* mutation were generated in the *TRα* knockout background (TRα^(−/−)^TRβ^(+/−)^) as described previously [27], and sexually mature TRα^(−/−)^TRβ∆19 ^(+/−)^ frogs were mated to produce tadpoles as described [29]. 

Mutations in *X. tropicalis* amidohydrolase domain-containing 1 (*AMDHD1*) were introduced through TALEN-mediated genome editing, and the TALENs targeting *AMDHD1* exon 3 were assembled as described [7,30]. In brief, the TALENs were designed by using TAL Effector-Nucleotide Targeter 2.0 (https://tale-nt.cac.cornell.edu, accessed on 21 December 2013) [31] and assembled as previously described [22]. The target sequences of AMDHD1-TALEN-ELD and -KKR were 5′-TTGTAGATGCACACACTCAC-3′ and 5′-TGCAAACTCGTGCACCCTGTC-3′, respectively. TALEN mRNAs were transcribed in vitro by using an mMESSAGE mMACHINE SP6 Transcription Kit (Thermo Fisher Scientific, Waltham, MA, USA) and injected into *X. tropicalis* embryos at the one-cell stage at 400 pg per embryo along with 200 pg of *DsRed* mRNA (Clontech, Palo Alto, CA, USA). The DsRed fluorescent signal was used to identify successfully injected embryos. The embryos with bright DsRed fluorescent signals were allowed to develop into sexually mature adult frogs (F0 generation frogs) and used to mate with wild-type frogs to produce tadpoles to screen for heterozygous mutant F1 germline animals. The heterozygous mutant F1 animals with a single-nucleotide deletion (AMDHD1-KO(∆1)) were further confirmed by PCR-cloning the target region into pCR2.1 TOPO vector (Thermo Fischer Scientific, Waltham, MA, USA) and sequencing and reared to adulthood (F1 heterozygous frogs). 

### 2.3. DNA Extraction and PCR Amplification

F2 generation tadpoles after onset of feeding (stage 45) were used for tail-tip clipping (3 to 5 mm) to obtain genomic DNA for genotyping as previously described [29]. The genomic DNA was subjected to first-round PCR with primer pairs encompassing the target region, common to both wild-type and mutant alleles (primer F and R for *MBD3* targets, TR_F and TR_R for *TRβ* targets, and A_F and A_R for *AMDHD1* targets) (Table 1) by using Taq DNA polymerase (Thermo Fischer Scientific, Waltham, MA, USA), and the products were diluted at 1:1000 to serve as templates for genotyping by using allele-specific primers in PCR or quantitative PCR (qPCR) reactions (Table 1). 

### 2.4. Genotyping through Competitive PCR with Dual Fluorescent Primers

Competitive PCR with dual fluorescent primers was essentially performed as described previously [28]. In brief, the IR700- and IR800-labeled primers (custom-designed and purchased from Integrated DNA Technologies, Coralville, IA, USA) specific for the mutant (IR700-Fm) and wild-type (IR800-Fwt) alleles (Table 1), respectively, were mixed with their respective unlabeled primers of same sequences at 1:50 for use in PCR reactions, with the final concentration of each primer at 0.5 μM. PCR reactions containing both the florescent primers and a common paired primer were performed with Taq DNA polymerase (Thermo Fischer Scientific, Waltham, MA, USA).

### 2.5. Genotyping through Quantitative PCR

For qPCR analysis, heterozygous mutant genomic DNA was amplified by PCR with the primer pairs encompassing the genome-editing target regions, common to both wild-type and mutant alleles, purified and serially diluted at 1:5 starting from 50 pg/μL concentration as standard DNA for quantification. Parallel qPCR reactions, with one using a wild-type-specific primer set and the other using a mutant allele-specific primer set, respectively, were performed on the same samples to quantify wild-type (Vwt) and mutant (Vm) alleles in each sample, respectively, by using the same standard DNA set for standardization. The qPCRs were performed by using PowerUP^TM^ SYBR^TM^ Green Master Mix on StepOne^TM^ Plus Real-Time PCR System (Thermo Fisher Scientific, Waltham, MA, USA). The Vwt and Vm were used to calculate the genotype index (iGenotype), defined as:

iGenotype = (Vwt − Vm)/(Vwt + Vm)

### 2.6. Gel Electrophoresis and DNA Detection

PCR products using unlabeled primers were resolved on an agarose gel, stained with ethidium bromide, and photographed with a digital camera attached to AlphaImager HP (ProteinSimple, Minneapolis, MN, USA). Fluorescent PCR products were denatured as previously described by mixing with an equal volume of 2 × Urea Loading Buffer (20 mM Tris-HCl, pH 8.0, 1 mM EDTA, 8 M urea, 0.05% Orange G), resolved on polyacrylamide gels (15% TBE-Urea PAGE Gel, Thermo Fischer Scientific, Waltham, MA, USA), and digitalized by scanning the gel on a LI-COR Odyssey Clx Scanner (LI-COR Biosciences, Lincoln, NE, USA) with the IR700 recorded as green and IR800 as red signals, and their respective signal strength for the PCR products of the heterozygous control were adjusted to be equal so the merged bands were shown as yellow for the control [28].

### 2.7. Statistical Analysis

Statistical analysis was performed through ordinary one-way ANOVA analysis with Dunnett’s multiple comparison test in GraphPad Prism 10 (GraphPad Software, San Diego, CA, USA).

## 3. Results

### 3.1. qPCR-Based iGenotype Indexes Are Distinctive for Different Genotypes

**Figure 1:**
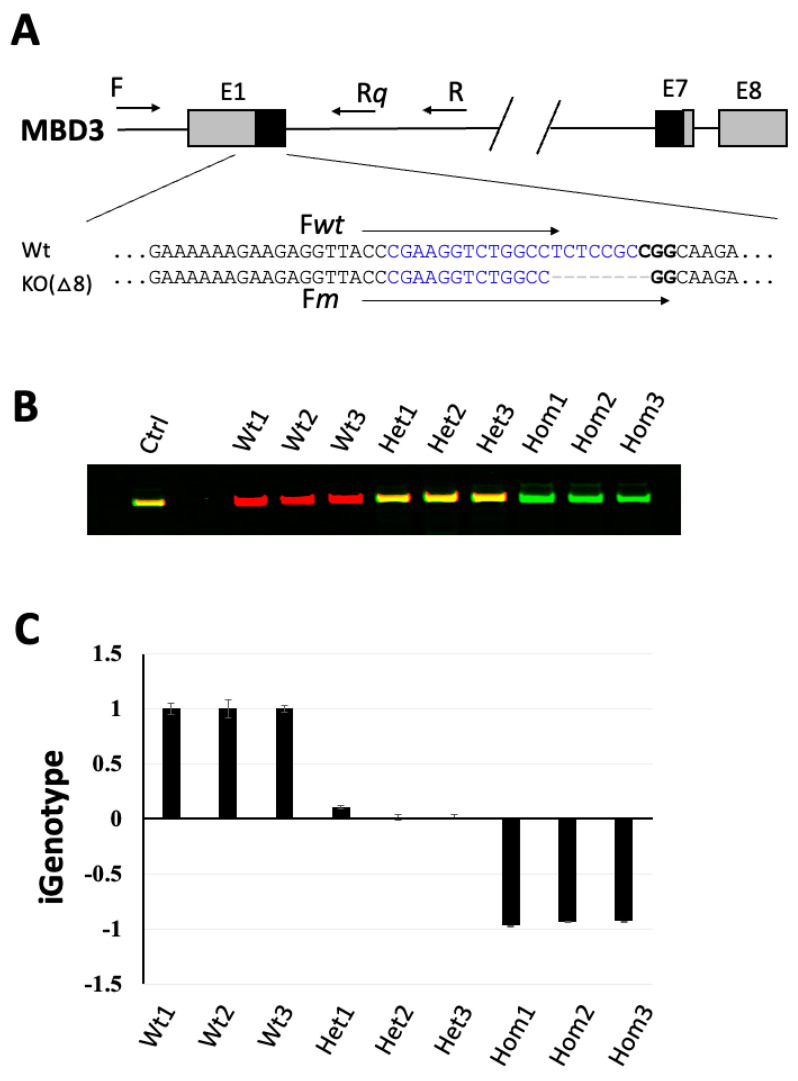
Comparison of qPCR-based iGenotype indexes for genotyping with the competitive PCR assay with dual fluorescent primers. Schematic illustration (**A**) showing wild-type and mutant *X. tropicalis MBD3* gene with an 8-nucleotide deletion (MBD3-KO(∆8)) and primers used in the parallel qPCR analysis to determine iGenotype indexes (Table 1). Genomic DNA from three animals of each genotype, i.e., the wild-type (Wt), heterozygous (Het), and homozygous (Hom) mutants, respectively, was subjected to the competitive PCR assay with dual fluorescent primers [28] (**B**) or qPCR-based iGenotype index analyses (**C**) for genotyping. Parallel qPCR reactions were performed in triplets, and the iGenotype indexes were plotted with mean ± standard deviations. Note that iGenotype indexes were converged to approximately +1, 0, and −1, respectively, for the wild-type, heterozygous, and homozygous mutants, respectively. E: exons; Ctrl: heterozygous control; F, Rq, R, Fwt and Fm: primers (Table 1). Wt: wild type; Het: heterozygous mutant; Hom: homozygous mutant.

We previous established a competitive PCR method with dual fluorescent primers to specifically and reliably genotype animals with point mutations or short indels [28], where IR800 and IR700 fluorescent dyes were used to label the wild-type-specific (IR800-Fwt) and mutant allele-specific (IR700-Fm) primers, respectively, to competitively amplify from the common targeted region by pairing with a common primer (R) in a single-tube PCR system (Figure 1A, Table 1). In this competitive PCR system, the heterozygous animal produced a yellow band, while the bands for the wild-type and the homozygous mutant were red and green, respectively (Figure 1B) [28]. In addition to the competition of allele-specific primers for their allele-specific targets that improves PCR specificity, another important step for the competitive PCR system to be successful is that red and green fluorescent signals should be adjusted to be equal for the heterozygous template (thus producing a yellow band), indicating the importance to apply the heterozygous template as the common reference to normalize the allele-specific PCR amplification efficiencies for the wild-type and the mutant alleles, respectively. This prompted us to hypothesize that, if we use the heterozygous templates as the common standard DNA for quantification of the allele-specific PCR signals in a single sample in parallel qPCRs, where one qPCR is for the wild-type allele and the other qPCR for the mutant allele, we should be able to obtain distinct allele-specific PCR values in the sample for identifying its genotype. To simplify the determination of the genotype, we introduced a genotype index (iGenotype, see Section 2), which would theoretically generate indexes of 1, 0, and −1, from wild-type, heterozygous, and homozygous mutant targets, respectively, if the allele-specific primers for parallel qPCRs work perfectly. To test this, we amplified the target region of heterozygous MBD3-KO(∆8) genomic DNA by using primers F and R (Figure 1A) and serially diluted the PCR products to serve as the common standard DNA in parallel qPCR reactions. The parallel qPCR was set up by using the same forward primers, except nonfluorescent, as used in the competitive PCR with dual fluorescent primers for genotyping above, to pair with a new common reverse primer, Rq (Figure 1A, Table 1, Fw was identical to IR800-Fwt and the Fm was identical to IR700-Fm in sequences, respectively), on genomic DNA samples of three individual animals for each genotype, i.e., wild-type, heterozygous, and homozygous MBD3-KO(∆8), respectively. The results indicated that the qPCR-based iGenotype indexes were calculated to be approximately 1, 0.02~0.1, and −0.93~−0.97, for the wild-type, heterozygous, and homozygous mutant templates, respectively (Table 2), and easily allowed the identification of the genotypes (Figure 1C), even though the wild-type-specific qPCR also produced significant signals on the homozygous mutant templates in the qPCR (Table 2). Thus, the allele-specific primers-based parallel qPCR system could be a reliable assay to determine genotypes without a need for fluorescent primers, microscope, and scanners. 

### 3.2. Genotype-Associated iGenotype Indexes Are Constant across Wide Ranges of Template Concentrations and PCR Primers, or Different Target Genes

**Figure 2:**
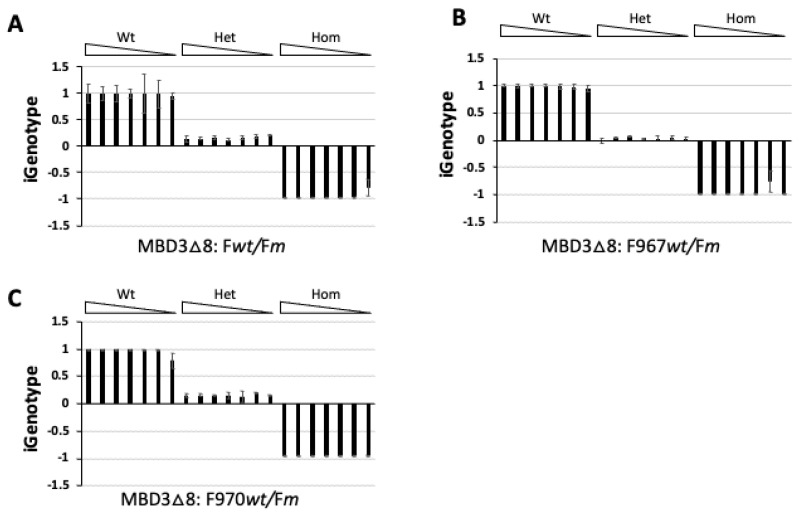
iGenotype indexes are highly reproducible and reliable for genotyping different allele-specific primer sets in parallel qPCR in a wide range of genomic DNA concentrations of MBD3-KO(∆8) tadpoles. The target regions of MBD3-KO(∆8) tadpoles were amplified by PCR with primers F and R (Figure 1A), purified and serially diluted at 1:5 to serve as templates in parallel qPCR analyses to determine the iGenotype indexes. The parallel qPCR reactions were performed in triplets with reverse primer Rq paired with either the MBD3-KO(∆8)-specific primer Fm for the mutant allele or in parallel with different wild-type-specific primer Fwt (**A**), F967wt (**B**), and F970wt (**C**) for the wild-type allele, respectively. The iGenotype indexes were plotted as mean ± standard deviation. Wt: wild type; Het: heterozygous mutant; Hom: homozygous mutant, 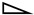: DNA concentration gradient.

Having shown that the qPCR-based iGenotype indexes could faithfully identify all three genotypes of a gene targeted by genome editing, we next asked if the accuracy of this genotyping method could be affected by template concentration, as DNA isolation from tissue biopsies can vary. To investigate this, we amplified the *MBD3* target region with primers F and R (Figure 1A) from wild-type, heterozygous, and homozygous MBD3-KO(∆8) mutant templates, respectively, purified and diluted the amplified DNA samples into 50 pg/μL each, then serially diluted them at 1:5 to serve as templates for the allele-specific qPCR analyses, as performed in Figure 1C. As shown in Figure 2A, the qPCR-based iGenotype indexes remained constant for each genotype within the wide concentration range tested for all three corresponding genotypes, suggesting that the qPCR-based iGenotype indexes could faithfully identify correct genotypes with great flexibility in template concentrations. To test if these observations apply to different primer sets in parallel qPCR analysis, we designed two alternative wild-type-specific primers, F967wt and F970wt (Table 1), to replace Fwt in qPCR paired with primer Rq, respectively, and evaluate the amounts of wild-type targets in the parallel qPCR analysis on the same samples used for Figure 2A, with the amounts of MBD3-KO(∆8) mutant targets that were still analyzed in qPCR using primers Fm and Rq. The results indicated that both alternative primers reproduced the same distinctive genotype-associated iGenotype indexes regardless of template concentrations (Figure 2B,C), suggesting that the iGenotype indexes for genotyping are flexible with primer designs.

**Figure 3:**
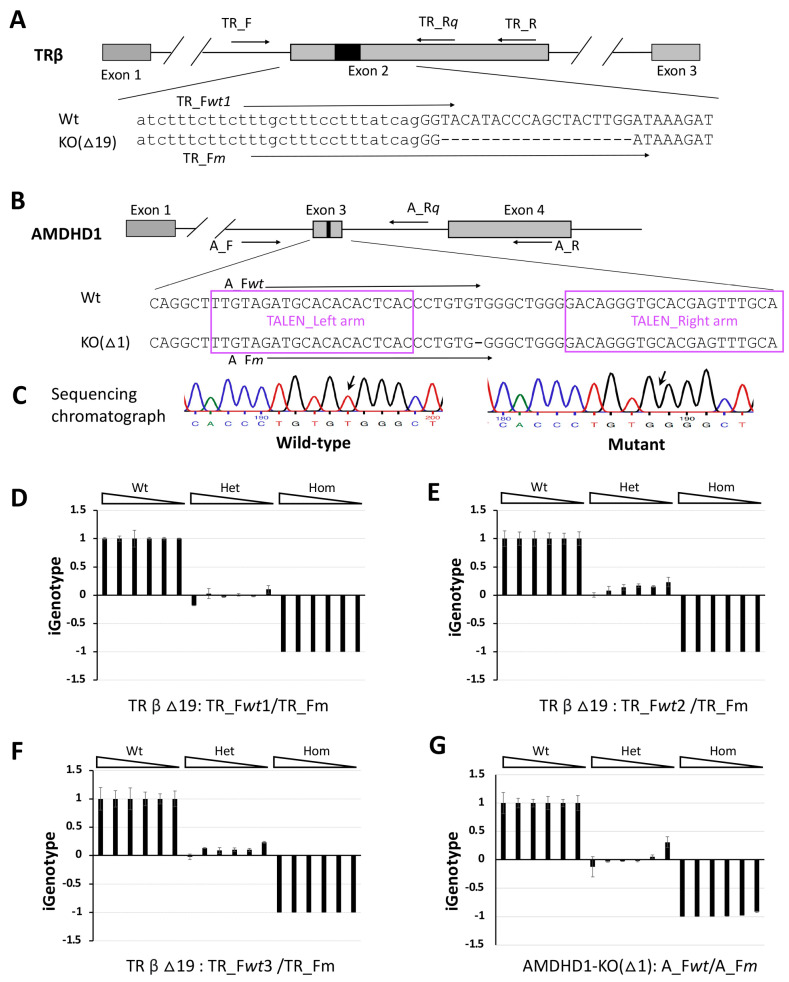
iGenotype indexes are highly reproducible and reliable for genotyping with different mutation targets in a wide range of genomic DNA concentrations. (**A**): Schematic diagram showing wild-type (Wt) *X. tropicalis* *TRβ* or a mutant with a 19-nucleotide deletion (TRβ∆19) and primers (TR_F, TR_Rq, TR_R, TR_Fwt1, and TR_Fm) used in the parallel qPCR analysis to determine iGenotype indexes. (**B**): Schematic diagram showing wild-type (Wt) *X. tropicalis* *AMDHD1* or a mutant with a single-nucleotide deletion (AMDHD1-KO(∆1)), which was generated through TALEN-mediated genome editing, and primers (A_F, A_Rq, A_R, A_Fwt, and A_Fm) used in the parallel qPCR analysis to determine iGenotype indexes. The TALEN-targeted regions were boxed and marked with TALEN_Left arm or TALEN_Right arm. (**C**): Sequencing chromatographs showing the wild-type (Wild-type) and AMDHD1-KO(∆1) mutant (Mutant) sequences around the single-nucleotide deletion (indicated by arrows). (**D**–**F**): The target regions of TRβ∆19 tadpoles were amplified by PCR with primers TR_F and TR_R, purified and serially diluted at 1:5 to serve as templates in the parallel qPCR analyses to determine the iGenotype indexes. The parallel qPCR reactions were performed in triplets with reverse primer TR_Rq paired with either TRβ∆19-specific primer TR_Fm for the mutant allele or in parallel with different wild-type-specific primer TR_Fwt1 (**D**), TR_Fwt2 (**E**), and TR_Fwt3 (**F**) for the wild-type allele, respectively. (**G**) The target regions of *AMDHD1* targets were amplified by PCR with primers A_F and A_R, purified and serially diluted at 1:5 to serve as templates in the parallel qPCR analyses, which were performed in triplets with reverse primer A_Rq paired with either AMDHD1-KO(∆1)-specific primer A_Fm for the mutant allele or in parallel with the wild-type-specific primer A_Fwt, to determine the iGenotype indexes. The iGenotype indexes were plotted as mean ± standard deviation. E: exon; -: deletion; Wt: wild type; Het: heterozygous mutant; Hom: homozygous mutant; 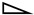: DNA concentration gradient.

To investigate whether iGenotype indexes for genotyping could be applied to other genes, we tested them on two different mutant *X. tropicalis* lines: one with a 19-nucleotide deletion in the *TRβ* gene (TRβ∆19, Figure 3A) and another with a single-nucleotide deletion in the *AMDHD1* gene (AMDHD1-KO(∆1), Figure 3B,C). We amplified the target region of an *X. tropicalis* TRβ mutant with a 19nt deletion (TRβ∆19) by using primers TR_F and TR_R (Table 1, Figure 3A) from templates of all three genotypes, respectively, purified and diluted into 4 pg/μL, then serially diluted at 1:5 to serve as templates in the parallel qPCR analysis, as done for the *MBD3* gene above. Similarly, three alternative forward primers for the wild-type allele (TR_Fwt1~3) and one forward primer for the mutant allele (TR_Fm) were designed to pair with a common reverse primer (TR_Rq), respectively, in the parallel qPCR system for genotyping. As expected, all iGenotype indexes were converged to approximately 1, 0, and −1, respectively, on all concentrations of respective wild-type, heterozygous mutant, and homozygous mutant templates (Figure 3D–F; Supplemental Data S1), just like what we observed for MBD3-KO(∆8) targets above. For AMDHD1-KO(∆1), we amplified the target region by using primers A_F and A_R (Table 1, Figure 3B) from templates of all three genotypes, respectively, purified the DNA and diluted it into 10 pg/μL, then serially diluted it at 1:5 to serve as templates in parallel qPCR analyses, again as done for the *MBD3* gene above. Due to limited choices of allele-specific primers differentiating the single-nucleotide deletion mutant, we designed one forward primer for the wild-type allele (A_Fwt) and one forward primer for the mutant allele (A_Fm), which had only a single different nucleotide primer sequence at the 3′-end, to pair with a common reverse primer (A_Rq), respectively, for the parallel qPCR system for genotyping. Consistently, all iGenotype indexes for the AMDHD1-KO(∆1) animals were converged to approximately 1, 0, and −1, respectively, on all concentrations of respective wild-type, heterozygous mutant, and homozygous mutant templates (Figure 3G; Supplemental Data S1). Taken together, these findings indicate that the genotype-associated iGenotype indexes can faithfully identify genotypes regardless of primer designs, template concentrations, or target regions with large or small indels including a single-nucleotide deletion, suggesting broad applications in genotyping genome-edited organisms.

### 3.3. Using Genotype-Associated iGenotype Indexes in Genotyping Animals

**Figure 4:**
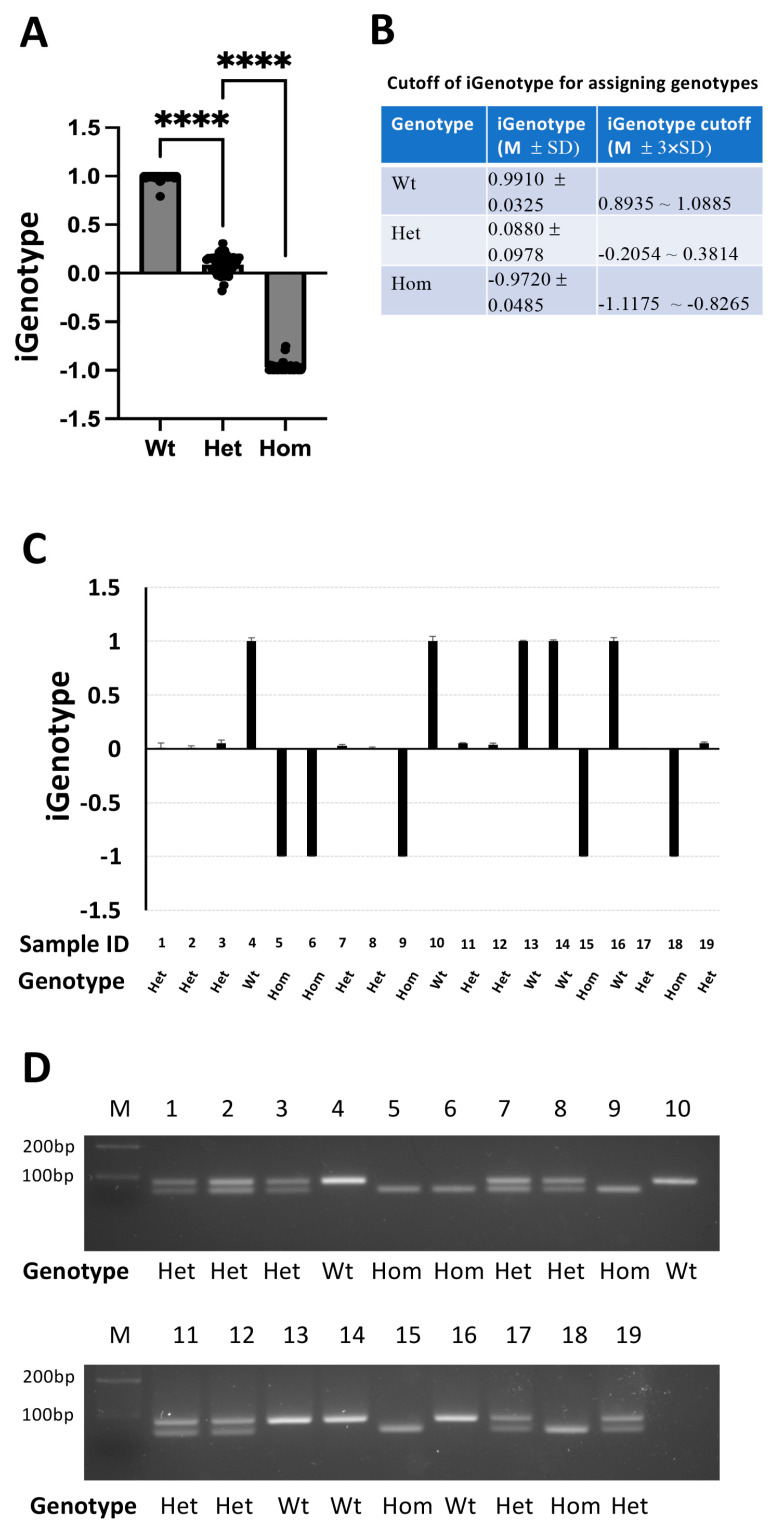
Genotyping animals with iGenotype indexes. The genotype-associated iGenotype indexes obtained from different combinations of primer sets in parallel qPCR reactions on different concentrations of MBD3-KO(∆8), TRβ∆19, and AMDHD1-KO(∆1) targets (Supplemental Data S1), respectively, were pooled together for statistical analysis with one-way ANOVA analysis (**A**) and used to establish the iGenotype index cutoff ranges for determining genotypes of unknown animals (**B**). To test the utilization of iGenotype index cutoff ranges for genotyping unknown animals, we mated heterozygous animals carrying the TRβ∆19 mutation. Genomic DNA was isolated from individual tadpoles for parallel qPCR reactions with TR-Rq paired with TRβ∆19-specific primer TR_Fm for the mutant allele and wild-type-specific TR_Fwt1, respectively, on 19 individual samples (**C**). The DNA was also used for genotyping with regular PCR with TR_Fa and TR_Ra (Table 1) for comparison (**D**), where the PCR products were resolved on a 3.5% agarose gel and photographed. The wild-type and mutant alleles had different sizes of PCR products and thus can be easily identified on the gene. Note that the genotypes identified based on iGenotype index cutoffs were all consistent with that from regular PCR genotyping. Wt: wild type; Het: heterozygous mutant; Hom: homozygous mutant; M: DNA marker. **** *p* < 0.001.

To use iGenotype indexes for genotyping animals of unknown genotypes, we next established genotype-specific cutoffs of iGenotype indexes for three different genotypes: wild-type, heterozygous, and homozygous animals. To determine the cutoffs, we combined all the experimental data from parallel qPCR analysis with different primer sets on varied DNA concentrations of MBD3-KO(∆8), TRβ∆19, and AMDHD1-KO(∆1) templates (Figure 2 and Figure 3, Supplemental Data S1) and calculated three respective genotype-specific means (M) and standard deviations (SD) of the iGenotype indexes. The data indicated that genotype-specific iGenotype indexes were distinct for the three genotypes (Figure 4A), with M ± SD at 0.9910 ± 0.0325, 0.0880 ± 0.0978, and −0.9720 ± 0.0485 for the wild-type, heterozygous, and homozygous mutants, respectively (Figure 4B). By using M ± 3 × SD as the cutoffs, we calculated the iGenotype index cutoffs that would be an iGenotype index greater than 0.8935 for the wild type, less than −0.8265 for the homozygous mutant, and between −0.2054 and 0.3814 for the heterozygous, respectively (Figure 4B). 

We next used these cutoffs to genotype animals generated from mating TRβ∆19 heterozygous frogs. We performed parallel qPCRs by using TR_Fwt1, TR_Fm, and TR_Rq to analyze some randomly selected tadpoles to identify their genotypes based on iGenotype indexes and compared the results with genotyping data by using regular PCRs with a pair of primers amplifying both the wild-type and the mutant targets (Table 1), which produces distinct sizes of PCR products for the wild-type and mutant alleles as resolved on an agarose gel [27]. The data indicated that the genotypes identified through the qPCR-based iGenotype indexes with the cutoffs (Figure 4C, Supplemental Data S2) were completely consistent with the genotypes identified through regular PCR analyses (Figure 4D), indicating that the statistically established iGenotype index cutoffs can be used to reliably identify genotypes of animals carrying mutations derived from genome editing.

## 4. Discussion

Genome editing as a major reverse genetic manipulation tool has broad applications in developmental and biomedical fields because of its powerful capacity to introduce mutations in targeted genes to study their function and the underlying molecular mechanisms. The key for successful utilization of such technologies is to have a faithful and simple approach for genotyping the germline mutations. Toward this, we established here a parallel qPCR-based approach to genotype germline *Xenopus* animals with mutations generated from genome editing and introduced an iGenotype index to simplify the identification of specific genotypes. The critical feature in our approach is that we used a standard DNA amplified from heterozygous mutant genomic DNA, which naturally contained equal ratios of wild-type and mutant alleles, in the parallel qPCR reactions to ensure that the iGenotype index converged to 1, 0, and −1 for wild-type, heterozygous, and homozygous mutants, respectively, to establish clear cutoffs for the specific genotypes. 

TALEN- or CRISPR/Cas9-mediated genome editing usually introduces wide varieties of mutations within the target region. PCR-based detection is convenient, economical, and widely used to detect mutations with large indels from genome editing that lead to significant size differences between the PCR products of wild-type and mutant alleles. However, the majority of the mutations introduced by genome editing are usually small indels or point mutations, limiting the use of PCR-based genotyping. Targeting a region harboring a restriction enzyme recognition site to be disrupted by genome editing [32], or selecting the mutant animal containing a new restriction enzyme recognition site introduced via genome editing, can expand application of PCR-based genotyping on animals with small indels, but this is costly and time-consuming because additional steps to purify and digest the PCR products are required prior to resolving the amplified DNA on gels, not to mention of the difficulty in finding such perfect target regions for genome editing or to screen for the mutant containing a new restriction enzyme recognition site. To overcome such limitations, PCR with allele-specific primers offers a promising approach to expand the application of PCR-based genotyping. However, this faces the difficulty of designing allele-specific primers with high specificity due to small indels or point mutations limiting the choices of primer selections, thus often leading to non-specific amplifications in PCRs and making this approach unreliable for genotyping. Many efforts have been made on improving the specificity of allele-specific PCR genotyping in recent years, and several strategies have been developed to genotype organisms with such small indels [33,34,35,36,37]; however, the practical use of those approaches is limited due to complex designs and/or lengthy assays.

*Xenopus* is an excellent model for diverse studies, including developmental and molecular biology, regeneration, and modeling human diseases [29,38,39,40]. *Xenopus* metamorphosis is a fascinating process during which the plasma thyroid hormone (T3) level peaks and adult organ development and maturation occur, involving extensive T3-dependent tissue remodeling in essentially all larval tissues [39]. Such a process offers a unique opportunity to identify and functionally characterize T3-regulated genes that potentially play critical roles in adult organ development, including organ-specific stem cell formations. We have previously identified and characterized many T3-regulated genes during *Xenopus* metamorphosis [39,41]. To functionally analyze such T3-regulated genes, we applied genome editing to knock out selected T3-regulated genes and established a two-color fusion protein assay to facilitate analyzing the mutation spectrum and efficiency of genome editing, from which we found that the resulting mutant tadpoles frequently contained short indels [21]. Thus, only in rare cases, we could use regular PCR genotyping based on the size differences of the amplified fragments from wild-type and mutant alleles [27]. In many cases, we relied on either PCRs with allele-specific primers for allele-specific amplification or DNA sequencing of PCR fragments to genotype the animals [22]. To improve the specificity and reproducibility of allele-specific amplification for genotyping, we recently developed a competitive PCR assay with dual fluorescent primers in a single-tube PCR system and demonstrated that this system could be used to reliably genotype animals harboring small indels even with imperfect allele-specific primer sets [28]. A drawback of this assay is that it requires fluorescent primers and a fluorescent microscopic scanner, which can prevent its applications when resources are limited. 

To overcome such limitations, we developed our current qPCR-based iGenotype index for genotyping by making use of two important features of the single-tube competitive PCR system: using allele-specific primers and normalizing amplification efficiency with allele-specific primers by using DNA from heterozygous animals as the control (i.e., adjusting the signals such that the amplification with primers for a wild-type allele is equal to that with primers for a mutant allele in PCR on a heterozygous DNA template). By using a parallel qPCR system in combination with the introduction of the iGenotype index, we found that the iGenotype indexes for wild-type, heterozygous, and homozygous mutant animals converged to 1, 0, and −1, respectively, even when some non-specific amplification occurred with allele-specific primers (Figure 1C, Table 2). This made it possible to use a simple qPCR procedure for easy and reliable genotyping over wide ranges of concentrations of DNA from tissue biopsies and different primer combinations, even when the allele-specific primers for a set of parallel qPCR analysis differ with only a single-nucleotide sequence at the 3′-end (Figure 1C, Figure 2A and Figure 3G, Table 1). The flexibility to design allele-specific primers for the parallel qPCR analysis thus provides a much better opportunity to void the interference of nucleotide polymorphism within the target region in designing the allele-specific primers. It thus overcomes the drawbacks and limitations of previous methods and offers an opportunity for automated analysis due to the numeric nature of the iGenotype index.

In summary, we have established a parallel qPCR system for easy genotyping by using heterozygous templates as the common standard references in both qPCRs. With the introduction of the qPCR-based iGenotype index that converged to 1, 0, and −1 for wild-type, heterozygous, and homozygous mutants, respectively, the method allows for quantitative analyses for genotype determination without a need for sophisticated equipment and reagents and can be potentially used for large-scale, automated genotyping. It should have broad applications in diverse settings in biomedical research and drug development and validation.

## Figures and Tables

**Table 1:** Primers for PCR and qPCR.

Gene	Primer ID	Genotype Specificity	Primer Sequences (5′-3′)	PCR Product
*MBD3*	F	Both	5′-CCGGCAGCGTGGTCCTGCTATC-3′	
	R	Both	5′-CTCTTTATTCCTCCAGCTGCACC-3′	645 bp
	IR800-F*wt*	wt	(IR800)5′-ACCCGAAGGTCTGGCCT-3′	315 bp *
	IR700-F*m*	m	(IR700)5′-ACCCGAAGGTCTGGCCG-3′	307 bp *
	R*q*	Both	5′-CTATAGTAATAGACATCGCTCT-3′	
	F*wt*	wt	5′-ACCCGAAGGTCTGGCCT-3′	50 bp **
	F*m*	m	5′-ACCCGAAGGTCTGGCCG-3′	42 bp **
	F967*wt*	wt	5′-GAAGGTCTGGCCTCTCC-3′	46 bp **
	F970*wt*	wt	5′-CCCGAAGGTCTGGCCTCT-3′	49 bp **
*TRβ*	TR_F	Both	5′-CTATCTAAAAGGACAACATTaga-3′	
	TR_R	Both	5′-GATATCACTATAACTGCTGTAGC-3′	246 bp
	TR_F*wt*1	wt	5′-TTGCTTTCCTTTATCAGGGTA-3′	140 bp ***
	TR_F*m*	m	5′-TTGCTTTCCTTTATCAGGGAT-3′	121 bp ***
	TR_R*q*	Both	5′-TCCACCTCCAATCTGTTACC-3′	
	F*wt*2	wt	5′-AGGGTACATACCCAGCTACT-3′	124 bp ***
	F*wt*3	wt	5′-AGGGTACATACCCAGCTACTT-3′	124 bp ***
	TR_F*a*	Both	5′-GGACAACATTAGATCTTTCTTTCTTTG-3′	87/68 bp (wt/m)
	TR_R*a*	Both	5′-CACACCACGCATAGCTCATC-3′	
*AMDHD1*	A_F	Both	5′-TGACCTCGCCCGACCTGTGGA-3′	688 bp
	A_R	Both	5′-TTGTTCCTGCTCTGAGCATTCTCTCT-3′	
	A_F*wt*	wt	5′-CACACACTCACCCTGTGT-3′	169 bp ^#^
	A_F*m*	m	5′-CACACACTCACCCTGTGG-3′	168 bp ^#^
	A_R*q*	Both	5′-AGACCAGGCAGGTATTTCTG-3′	

*: pair with common reverse primer R; **: pair with common reverse primer Rq; ***: pair with common reverse primer TR_Rq; ^#^: pair with common reverse primer A_Rq. Both: wild type and mutant; wt: wild type; m: mutant.

**Table 2:** qPCR-based iGenotype index faithfully identifies all three genotypes reliably.

Sample ID	Value_wt		Value_m		iGenotype	
	Mean	SD	Mean	SD	Mean	SD
Wild type1	70,726	3405.8	1.3247	0.13861	0.99996	0.04815
Wild type2	5948.0	501.85	0.10228	0.00202	0.99997	0.08437
Wild type3	8872.8	246.82	0.16042	0.00810	0.99996	0.02782
Heterozygous1	13,443	383.77	10,951	253.05	0.10212	0.01573
Heterozygous2	14,239	720.72	13,771	528.56	0.01669	0.02573
Heterozygous3	12,497	438.65	12,081	606.20	0.01694	0.01785
Homozygous1	64.617 *	2.9061	4004.7	41.622	−0.96824	0.00071
Homozygous2	139.13 *	7.5388	4213.1	723.54	−0.93606	0.00173
Homozygous3	665.56 *	27.653	18,307	540.69	−0.92984	0.00146

*: The wild-type-specific qPCR primers amplified non-specifically on the homozygous mutant templates significantly (in bold). However, the iGenotype index still accurately genotyped the animals. SD: standard deviation.

## Data Availability

Data are contained within the article and Supplementary Materials.

## Supplementary Materials

The following supporting information can be downloaded at: https://www.mdpi.com/article/10.3390/cells13030247/s1, Supplemental Data S1: iGenotype indexes from parallel qPCR using different primer sets on different concentrations of varied templates; Supplemental Data S2: Parallel qPCR-based iGenotype indexes for the *X. tropicalis* animals with a thyroid hormone receptor beta (TRβ) mutation of 19-nucleotide deletion (TRβ∆19); and Original gel imagines.pptx.
