# Supplementary material for: Simplifying Genotyping of Mutants from Genome Editing with a Parallel qPCR-Based iGenotype Index
Source: Cells. 2024 Jan 29;13(3):247. doi: 10.3390/cells13030247 (PMC10854663; doi:10.3390/cells13030247)
Supplement: Supplementary file 1 [file cells-13-00247-s001.zip › Original gel imagines.pptx]

## Slide 1
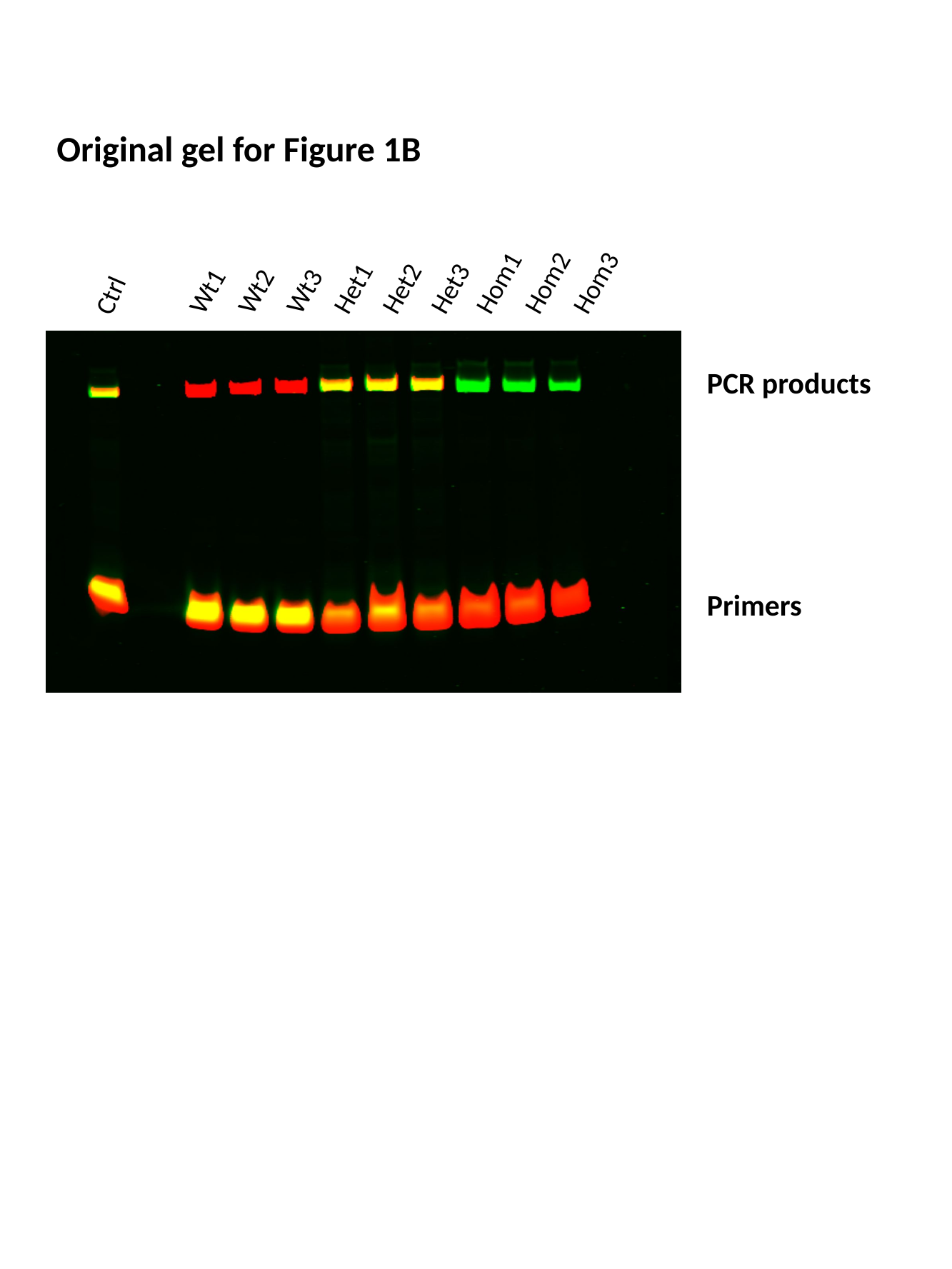

Original gel for Figure 1B
Hom1
Hom2
Hom3
Het1
Het2
Het3
Wt1
Wt2
Wt3
Ctrl
PCR products
Primers

## Slide 2
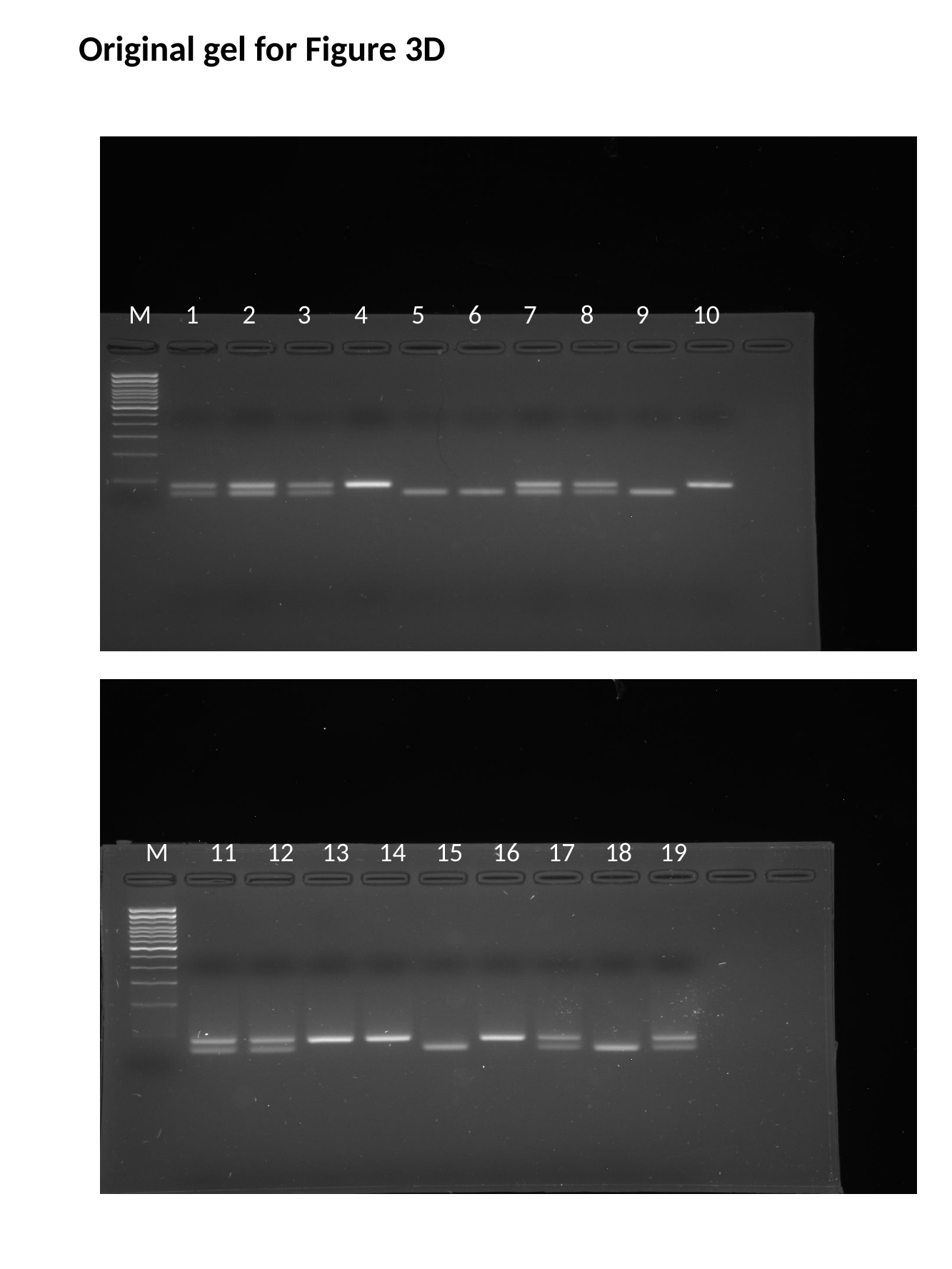

Original gel for Figure 3D
M
1
2
3
4
5
6
7
8
9
10
M
11
12
13
14
15
16
17
18
19
